# Equivalent spin-orbit interaction in the two-polariton Jaynes-Cummings-Hubbard model

**DOI:** 10.1038/srep11945

**Published:** 2015-07-10

**Authors:** C. Li, X. Z. Zhang, Z. Song

**Affiliations:** 1 School of Physics, Nankai University, Tianjin 300071, China; 2 College of Physics and Materials Science, Tianjin Normal University, Tianjin 300387, China

## Abstract

A cavity quantum electrodynamics (cavity-QED) system combines two or more distinct quantum components, exhibiting features not seen in the individual systems. In this work, we study the one-dimensional Jaynes-Cummings-Hubbard model in the two-excitation (two-polariton) subspace. We find that the centre momentum of two-excitation induces a magnetic flux piercing the equivalent Hamiltonian *H*_*k*_ in the invariant subspace with momentum *k*, which can be described as a 4-leg ladder in the auxiliary space. Furthermore, it is shown that the system in *π*-centre-momentum subspace is equivalent to a lattice system for spin-1 particle with spin-orbit coupling. On the basis of this concise description, a series of bound-pair eigenstates which display long-range polaritonic entanglement is presented as a simple application.

A cavity quantum electrodynamics (cavity-QED) system combines two or more distinct quantum components, exhibiting features are not seen in the individual systems. Such a system offers a promising platform from which to study novel quantum phenomena. The Jaynes-Cummings-Hubbard (JCH) model is an archetype of such hybridization, which consists of the Jaynes-Cummings (JC) model and the coupled cavities. The JCH model was proposed to exploit the atom-light interaction in coupled microcavity arrays to create strongly correlated many-body models^1^,^2^,^3^,^4^,^5^, though it has been studied in a variety of directions. In the context of quantum simulation, several good quantum simulators have been proposed that realize the JC model, a vital component of the JCH model, such as superconducting circuits (see the review^6^ and references therein).

Previous studies have mainly focused on the ground state phase of many-particle systems and the dynamics in a single-particle system. The Mott insulator phase and superfluid phase are identified by the traditional order parameter. For example, the average of the annihilation operator^3^ and observable quantities such as atomic concurrence and photon visibility^7^. Studies of single-particle dynamics suggests that this hybrid architecture can parallel a coherent quantum device to transfer and store quantum information as well as to create laser-like output^8^,^9^,^10^. Recently, the few-body problem for the JCH Hamiltonian has also been investigated^11^,^12^, postulating the existence of two-polariton bound states when the photon-atom interaction is sufficiently strong.

In this work, we study the one-dimensional JCH model in the two-excitation (two-polariton) subspace. In each invariant subspace, the sub-Hamiltonian is equivalent to a 4-leg ladder with an effective flux which is proportional to the centre momentum of two excitations. It is shown that in *π*-centre-momentum subspace, the ladder system can be reduced to a lattice system of spin-1 particles with spin-orbit coupling. On the basis of this concise description, a series of bound-pair eigenstates, which display long-range polaritonic entanglement is presented as a simple application.

## Results

### JCH Model

The JCH model describes a cavity array doped with two-level atoms in which every cavity is embedded by a single two-level atom. In this model, the dipole interaction leads to complex dynamics involving photonic and atomic degrees of freedom, which is in contrast to the widely studied Bose-Hubbard model. Such a cavity-QED system can be implemented with a defect array in a photonic crystal^13^,^14^ or a Josephson junction array in a cavity^8^,^15^,^16^. The Hamiltonian of a cavity-QED system, or indeed a lattice atom-photon system


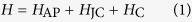


can be written as three parts: free Hamiltonians of the atom and photon,


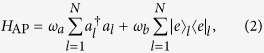


the JC-type cavity-atom interaction in the *l*-th defect,


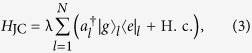


(with strength λ) and the photon hopping between nearest neighbour cavities,


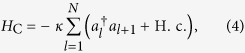


where *κ* is the hopping integral constant for the tunnelling between adjacent cavities. Here 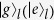 denotes the ground (excited) state of the atom placed at the *l*-th cavity, and 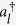 and *a*_*l*_ are the creation and annihilation operators of a photon at defect *l*. The rotating-wave approximation, which requires that 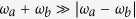 and 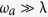, is satisfied automatically in the JCH model. Obviously the total excitation number


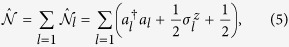


is a conserved quantity for the Hamiltonian *H*, i.e. 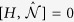, where 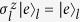 and 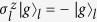. Here the excitation refers to a combination of photonic and atomic excitations, termed as polaritons^2^. Therefore 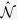 is the excitation number of the polaritons. For each cavity, the basis state can be expressed as 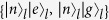, where the basis state of the Fock space for the *l*-th cavity is 
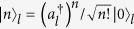
. In this paper, we consider the invariant subspace with 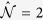, which is spanned by a basis in the form


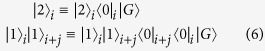



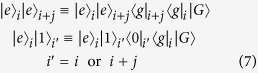


where 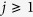 and 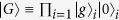 denotes the empty state with zero 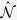. We denote the matrix representation of the Hamiltonian of Eq. (1) in the basis of Eq. (6) as 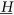. In the case of real values of *κ* and λ, we have 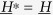, which indicates that 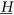 has time-reversal symmetry.

### 4-Leg Ladder with flux

The system is translational invariant^17^. In the two-particle Hilbert space, the Hamiltonian *H* can be written as 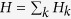 with periodic boundary conditions, where


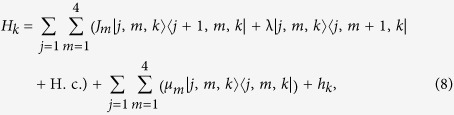


and


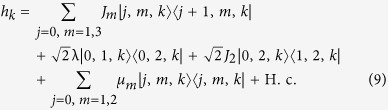


Here the set of states 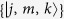 is defined as following: For *j* ≥ 1, it reads


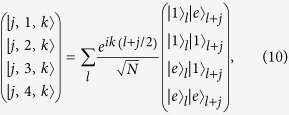


and


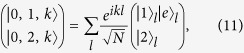


where we have taken 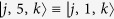 for *j* ≥ 1, and 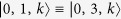. The parameters read


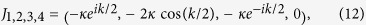


and


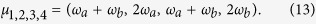


**Figure 1:**
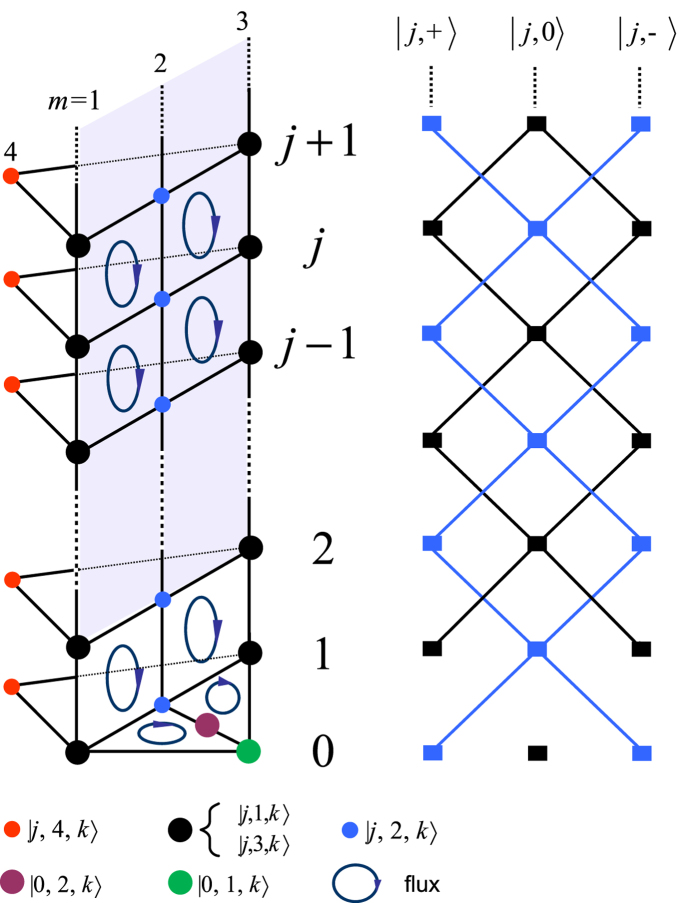
Schematic of the structures of equivalent Hamiltonians for the one-dimensional JCH model with two polaritons. (**a**) In the invariant subspace with centre momentum *k*, the equivalent Hamiltonian *H*_*k*_ describes a 4-leg ladder with *k*-dependent flux. The shadow indicates the semi-infinite uniform ladder. (**b**) For *k* = *π*, *H*_*k*_ is equivalent to a spin-1 chain with spin-orbit interaction. The graph of *H*_*π*_ consists of two unconnected subgraphs, characterized by the parity Π = ±1. *H*_*k*_ indicates that *H*_*π*_ can be further decomposed into two independent parts *H*_*o*_ (dark) and *H*_e_ (blue).

The expression of *H*_*k*_ in Eq. (8) has a clear physical meaning: 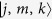 denotes the site state for the *j*-th site on the *m*-leg of a 4-leg ladder system with the effective magnetic flux piercing the plaquette. The flux is proportional to the centre momentum of two excitations. The structure of *H*_*k*_ is schematically illustrated in Fig. 1. We note that the matrix representation of *H*_*k*_ in the basis of Eqs. (10) and (11), *H*_*k*_, breaks the time-reversal symmetry. Nevertheless, we still have 
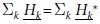
, as 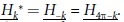 In essence, the nonzero plaquette flux arises from the relationship between the complex coupling constants 
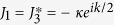
. In contrast, one can see from *H*_*k*_ that the complex λ cannot induce a nonzero plaquette flux. We would like to stress that the effective magnetic field in the present model is intrinsic, not depending on an external control, but relying on the value of *k*. We note that there are two kinds of excitations, the spin-up (excited atom) state and a photon, which obey two different statistics (that for hardcore bosons and bosons). This may be the origin of the equivalent plaquette flux. Then, the underlying mechanisms for obtaining the equivalent plaquette flux in our work and that of Ref. 18, 19 are different.

In order to understand the mechanism of the effective flux, we investigate the exchange process for photon and atomic excitations beginning in the state 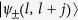, passing through 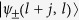, returning back to 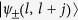, where


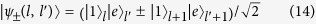


are states in a different invariant subspace. The action of *H* provides a loop for this task:


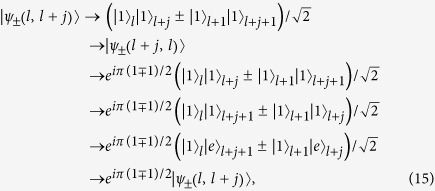


which correspond to a ring network with six vortices. It shows that this round trip acquires a phase 0 or *π*, which is equivalent to the effect of a flux piercing the loop. We note that the flux depends on the sign ± in each of the states. On the other hand, the sign ± in state 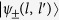 indicates the symmetry or antisymmetry of the state under the transformation 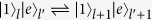. This investigation implies that the origin of the effective magnetic field may be the special statistical properties of two quasi-particles in each invariant subspace.

### Equivalent Hamiltonian in *π*-momentum subspace

We focus on the case k = *π* and *ω*_*a*_ = *ω*_*b*_, which leads to 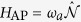. It is a simple but non-trivial case, since the hopping along leg 2 is switched off but the plaquette flux still exists. We note that the on-site potentials *μ*_*l*_ of different legs are identical, which allows us to ignore the diagonal terms in *H*_*π*_.

Introducing the three-dimensional vector bra and ket for


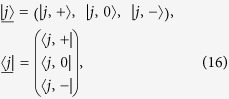


the Hamiltonian *H*_*π*_ in the *π*-momentum subspace can be expressed as


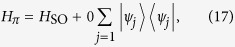


with 
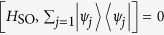
, which indicates that *H*_*π*_ is block-diagonal.

Here 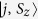 represents a spin-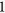 particle at the *j*-th site with spin polarization *S*_*z*_ = 0, ±1 defined as


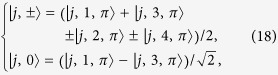


for *j* ≥ 1, and


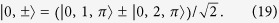


In addition, the state 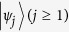 is defined as


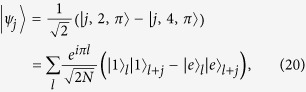


which, together with states 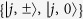, constructs the complete orthogonal set. Of particular interest, 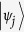 is the eigenstate of *H* with energy 2*ω*_*a*_. In Eq. (17), the zero-energy term represents this point, where we have ignored a constant shift 2*ω*_*a*_.

The sub-Hamiltonian *H*_so_ is in the form


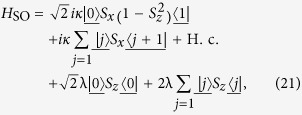


where the Pauli spin matrices for a spin-1 particle are given by


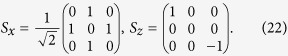


Consequently, within a specific invariant subspace, a system made of *N*-cavity array with a single two-level atom embedded in each cavity appears to be equivalent to a tight-binding chain of spin-1 particle with spin-orbit interaction. The structure of *H*_so_ is schematically illustrated in Fig. 1. Intuitively, the graph of *H*_so_ consists of two unconnected subgraphs. This can be clarified by observing that the parity operator


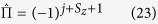


where 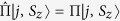 and 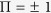 characterize the two subgraphs.

We can thus conclude that the equivalent Hamiltonian *H*_so_ can be decomposed into two independent parts


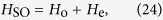


with [*H*_o_, *H*_e_] = 0, and 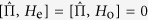. The sub-Hamiltonians are defined as


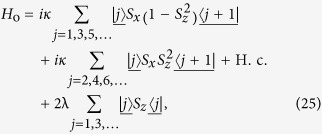


and


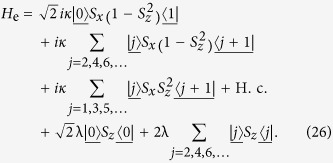


The subscripts o and e represent the contributions associated with the sites of odd and even parity Π. The structures of *H*_o_ and *H*_e_ are schematically illustrated in Fig. 1. This figure indicates that the invariant space with *k* = *π* is split in two unconnected subspaces. This allows us to investigate the Hamiltonians *H*_o,e_ separately.

### Exact bound-pair states

Based on the above analysis, besides states 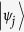, one can also construct a series of bound-pair states of the form


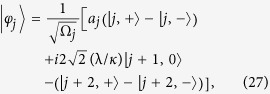


where the normalization factor Ω_*j*_ and amplitudes *a*_*j*_ are given as


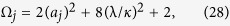


and


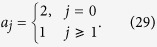


A straightforward derivation shows that


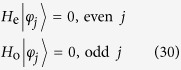


i.e. 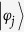 is an eigenstate of *H*_so_. This is a direct application of the bound state theorem given in^20^, which states that any eigenstate of a sub-graph is also an eigenstate of the whole, if the nodes cover all the joint points. We are interested in the expression of these states in the atom-photon basis, given by
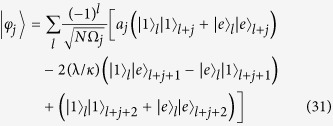
for *j* ≥ 1, and


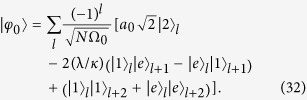


Alternatively, a direct derivation can check our conclusion for the original Hamiltonian of a lattice atom-photon system in Eq. (1) that


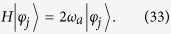


The formation mechanism of these bound-pair eigenstates can be understood as the result of quantum interference, which is presented in the Methods section.

### Long-range polariton-polariton entanglement

We now study the features of the obtained eigenstates. It is apparent that the pair states 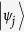 and 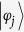 are entangled states. In the strong coupling limit 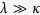, we have


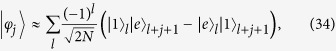


which is the superposition of entangled states between two cavities at distance *j* + 1. States


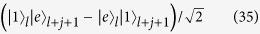


in 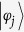 and


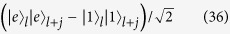


in 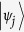 are both maximally entangled states of the *l*-th and (*l* + *j*)-th (or (*l* + *j* + 1)-th) cavities for the two modes: excited cavity fields and excited atom modes. To demonstrate this concept in a precise manner, we introduce lower branch and upper branch exciton-polariton states,


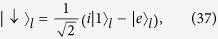



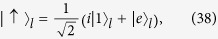


the superposition of which yields a polariton qubit state at cavity *l*. As 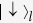 and 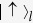 are a basis, it is given that


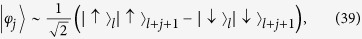



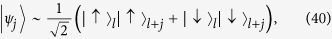


which are standard Bell states. We see that the entanglement does not decrease as the distance *j* increases. This entanglement is one of great importance in the new field of quantum information theory. Polaritons^21^, as quasiparticles of light and matter, are the most promising solution for the interface between electronic and photonic qubit states. However, there is another form of entanglement: the atomic entanglement, which is based on the atomic qubit, referring to ground and excited atomic states. This is different from the entanglement discussed above, two atoms with state 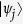 (or 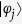) in the 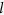-th and (*l* + *j*)-th (or (*l* + *j* + 1)-th) cavities do not entangle with each other, which means that atomic entanglement does not exist in our model. A demonstration of this will be given in the Methods section.

## Discussion

In summary, we have established the link between the two-excitation JCH model and the single-particle 4-leg ladder with an effective flux, which has proven to be equivalent to a chain system of spin-1 particle with spin-orbit coupling. This study also introduces a mechanism to construct a series of bound-pair eigenstates which display long-range polaritonic entanglement. This finding reveals that cavity-QED systems can offer rich features and useful functionality, which will motivate further investigation.

However, it is a great challenge to realize the predictions in experiment. Although the obtained results do not require a special range of system parameters, several issues should be concerned for an experimental realization of our findings. Firstly, JCH model is obtained under the rotating wave, single-mode, and narrow band approximations. Then, parameters in a real system should be in the range to meet the condition of such approximations. Secondly, one needs a scheme for the preparation of the bound states. Below there is a possible way based on the fact that the proposed eigenstates have the same energy 2*ω*_*a*_, which is identical to the eigen energy of an uncoupled system, i.e., *κ* = λ = 0. (i) Generating extended state of two photons for the system with λ = 0 but *κ* ≠ 0. This is relatively accessible since all the eigenstates of such a system are extended states. (ii) Taking *κ* → 0. Then all two-photon extended states have energy 2*ω*_*a*_. (iii) Switching on and increasing λ and *κ* slowly. The initial state should evolve to the states with excited atomic state. There should be a large probability for the transition between states with the same energy, including our target states. This scheme requires a temporal control of parameters λ and *κ* in experiment. At this stage, this is just a qualitative analysis, but will motivate further quantitative investigation for the procedure of entangled state preparation in a cavity-QED system.

## Methods

### Construction of bound-pair eigenstates

The formation mechanism of these bound-pair eigenstates can be understood as the result of quantum interference in the following three process.

- (i) We start with the case of switching off the JC interaction such that λ = 0. The atoms are decoupled from the cavity array. It is uncomplicated to check that

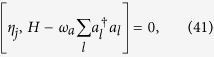


where the operator *η*_*j*_ is defined as


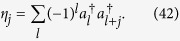


According to a similar analysis in Ref. 22, it is found that state


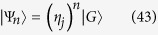


is an eigenstate of *H*,


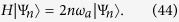


Furthermore, it is worthy to note that even for a Bose Hubbard model, which involves the on-site interaction


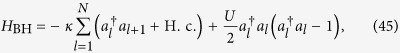


we still have


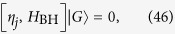


which leads to the conclusion that 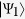 is an eigenstate of *H*_BH_.

The essence of the construction of 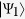 is due to the destructive interference between the two transitions from states 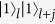 and 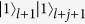



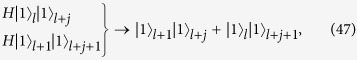


which results in


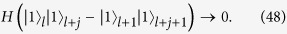


Here the contribution of *H*_0_ is ignored. We refer to this as a Hubbard-type process.

- (ii) Now we consider the case of switching off the tunnelling between cavities, *κ* = 0. Each cavity becomes separated from its neighbours. We have the identity

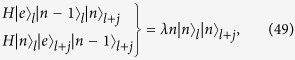


which results in


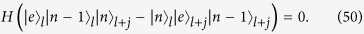


This means that there is destructive interference between the two paths, which are the atom-photon transitions in the two different cavities *l* and *l* + *j*. It is a pure QED process in a JC model, which is referred as a JC-type process. It is easy to check that the combination of Hubbard and JC-type processes results in the formation of the eigenstate 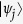.

- (iii) The crucial process that makes state 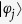 become an eigenstate of the complete Hamiltonian is a combination of the above two processes (i) and (ii). In this case, the excitation number must be 2. The transitions which result in destructive interference are

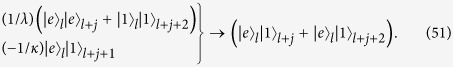


**Figure 2:**
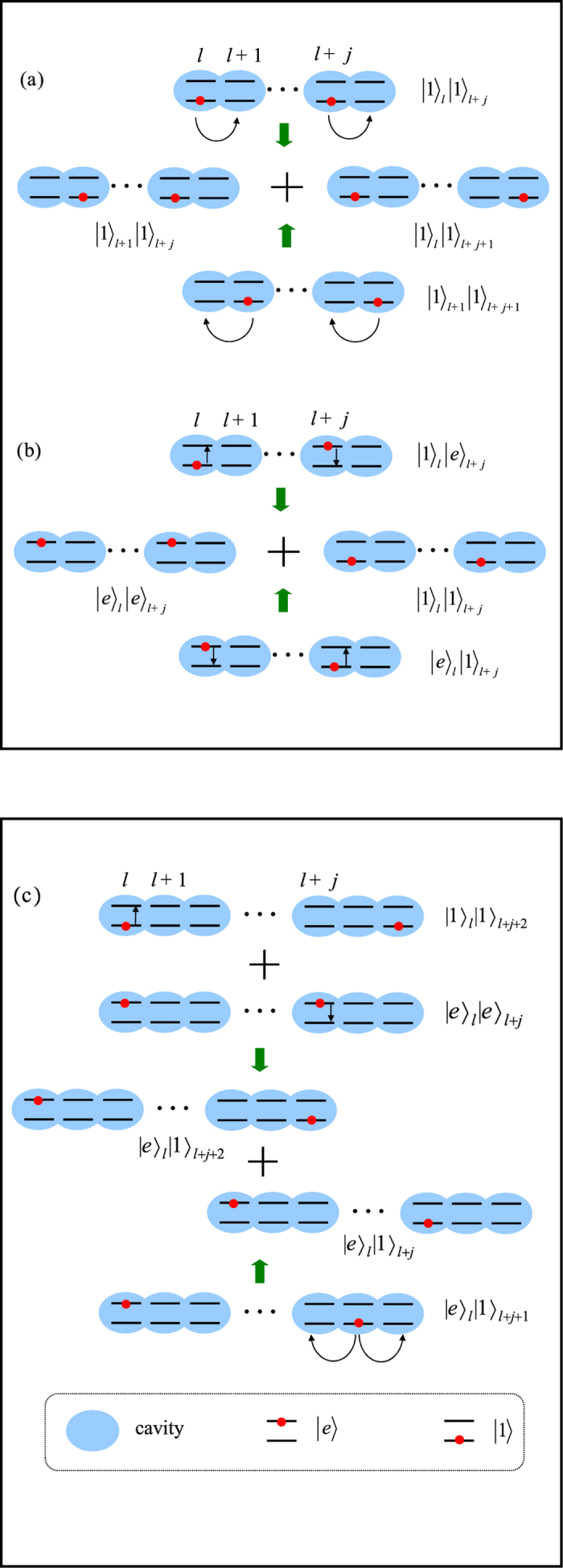
Schematic illustration for the mechanism of the formation of bound pair eigenstates. There are three types of destructive interference processes which result in the exact eigenstate 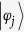. (**a**) The Hubbard-type process represented in Eq. (47). (**b**) The JC-type process represented in Eq. (49). (**c**) The key process referred to as mixed-type in Eq. (51) shows that the cancellation of the transitions requires an optimal ratio between the parameters λ and *κ*.

We find that the cancellation occurs only if the amplitudes of the two components 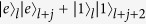 and 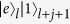 are properly assigned. We refer to this as the mixed-type process. In Fig. 2, three the processes for the formation mechanism of the bound pair state are schematically illustrated.

### Atomic entanglement

The atomic entanglement can be characterized by concurrence^7^. The reduced density matrix for two atoms in the *l*-th and (*l* + *j* + 1)-th cavities is


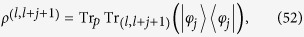


where Tr_*p*_ denotes the trace over all photon variables and Tr_(*l*,*l*+*j*+1)_ denotes the trace over all atomic variables except for the *l*-th and (*l* + *j* + 1)-th atoms. It has been shown in Ref. 7 that the formula for the concurrence of two quasi-spin particles in a hybrid system is the same as that for a pure spin-1/2 system^23^,^24^,^25^. Then, the concurrence 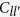 shared between two atoms *l* and *l*′ is obtained as


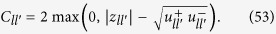


in terms of the quantum correlations


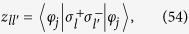



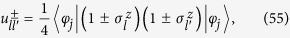


where 
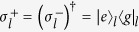
. It is a straightforward calculation to show that 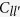 is always zero for both states 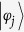 and 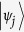.

## Additional Information

**How to cite this article**: Li, C. *et al.* Equivalent spin-orbit interaction in the two-polariton Jaynes-Cummings-Hubbard model. *Sci. Rep.* **5**, 11945; doi: 10.1038/srep11945 (2015).
